# Picogram-Scale Interstellar Probes via Bioinspired Engineering

**DOI:** 10.1089/ast.2022.0008

**Published:** 2022-12-06

**Authors:** George Church

**Affiliations:** Wyss Institute, Harvard University, Boston, Massachusetts, USA.

**Keywords:** Interstellar probe, Synthetic biology, Breakthrough Starshot

## Abstract

For exploring nearby stars, let us consider the challenges of a picogram- to nanogram-scale probe to land, replicate, and produce a communications module based on biominerals at the destination. A billion such probes could be launched for similar cost as a single gram-scale probe. One design is a highly reflective light sail, traveling a long straight line toward the gravitational well of a destination star, and then photo-deflected to the closest nonluminous mass—ideally a planet or moon with exposed liquid water.

## 1. A Brief Note on Terminology

The term Microsatellite or “microsat” is usually applied to an artificial satellite with a mass between 10 and 100 kg, nanosatellites between 1 and 10 kg, and femto-satellites <100 g. The lightest so far is 33 g (Izquierdo and Tristancho, 2011). The Breakthrough Starshot manifesto says “Nanocrafts are gram-scale robotic spacecrafts” (Siegel, 2022). This article simply refers to the mass of probes in grams and compares the proposed pico/nanogram-scale landing probes with previous “replicating” probe proposals and with gram-scale Starshot flyby proposals.

## 2. Previous Proposals for Replicating Probes

“In June of 1948 von Neumann gave three lectures … [which] contained the most detailed description of the parts of his self-reproducing automaton… eight kinds of parts … stimulus organ… inhibitory organ…coincidence organ …stimuli producer … rigid member … fusing organ … cutting organ … muscle” (von Neumann and Burks, 1966). These concepts were pathbreaking but have remained far from practical implementation. Freitas (1980) described “a self-reproducing starprobe … with generation time ∼10^3^ years” with 84 chemical elements explicitly assigned “mass flow requirements for each element,” and masses of replicating and transporting units estimated at millions of kilograms. The recent work from Haliki (2020) and Osmanov (2020) describe von Neumann probes quite abstractly with replication based on hydrogen collisions, with a doubling time of 9 years.

In 1978, a prescient proposal was made to “send biological machine seeds … moving at near-to-light speed … negligible risk of meteoritic impact … being so tiny, inertial effects would be easily overcome… a major technological problem would be to slow the projectiles down on arrival … converting some of the mass into laser…to help produce a braking effect … on arrival at a biologically suitable planet, this seed would start to grow producing all the instruments …” (Davies, 1978).

In 2016, the Genesis project proposed a “low-cost robotic microcraft equipped with an on-board gene laboratory for the *in situ* synthesis of the microbes… accelerate lightweight interstellar probes with … arrays of powerful lasers … Decelerating … using magnetic and/or electric sails…Seeding the candidate planet with *in situ* synthesized lifeforms” (Gros, 2016).

No estimates were given for the “low-cost” or “lightweight,” but even huge improvements on current biochemical synthesis robots would be far more than gram scale. No mention was made of replication of the full probe or von Neumann probes, nor why they synthesize microbes *in situ* rather than simply bringing a diverse set. They felt it “irrelevant how long it would take for a Genesis craft to arrive to the target. A few millennia more or less.” The attendant significant radiation damage was not mentioned.

In 2020, the Initiative for Interstellar Studies (i4is) proposed a “70% self-replicable probe based on current and near-term technologies … could be launched within the next 10 years, if microchips and other complex electronic components are brought with the initial probe and are not replicated” (Borgue and Hein, 2020). In contrast to the project above, the i4is proposal did not mention living beings, but does make cost and mass estimates, with a 100-kg probe (not counting the mass of stored components for replicated probes) with 18 m^2^ of solar panels and “utilization of a launcher such as the Atlas V, with launch cost of 14k$/kg.” As with the Genesis Project, the i4is project was not aimed at relativistic speeds.

## 3. Breakthrough Starshot Gram-Scale Probe (and Potential for Probe Size Reduction)

In contrast to the proposals above, the 2016 Starshot proposal (Lubin, 2016) aimed for a flyby, not a landing. The goal is the planet Proxima Centauri b, 4.73 light years away at a speed of 20% light speed (0.2c). The payload includes cameras, thrusters, power, navigation, communication, and meter-scale light sails. Minimizing capital cost (to $8.0 billion) for the ground-based beam director, the energy needed to accelerate each sail costs $6M for a 4.1-m diameter sail accelerated for 9 min (for a 3.6-g probe, 200 GW max. transmitted power) (Parkin, 2018). If the payload were picogram to nanogram scale, then the capex might be reduced more than linearly (*i.e.,* even better than billion fold) due to the lower complexity of a simple infrared (IR) optics relative to the “filled array of telescope elements that form a 2.7-km effective diameter.”

The lowered mass and power needs might permit launches from 30 to 50 km high balloons or >160-km-high Low Earth Orbit satellites, thereby reducing atmospheric drag and light attenuation through absorption and scattering. For less than the cost of 1000 launches at gram scale, one might get a trillion nanogram launches or 10^15^ picogram-scale launches. Admittedly, one might need that many to get even a few to land in a location permissive to building communication with the Earth. Most living cells on the Earth are picogram scale and yet perform functions, such as replication from only simple chemical inputs, impossible for all current human-made machines.

## 4. Acceleration and Deceleration

Heller and Hippke (2017) noted that “stellar photon pressures of stellar triple α Cen A, B, Proxima+gravity assists … maximum injection speed to park a sail with mass-to-surface ratio (σ) similar to graphene (7.63 × 10^−4^ g/m^2^) in orbit around Proxima is 4.6%c … to carry a payload of 10 g is about 10^5^ m^2^” (a 76-g sail). Since the maximum velocity is proportional to (A/m)^0.5^, a payload of a picogram cell might require a sail of 10^−8^ m^2^ (7.6 pg). (They also note that Sirius A, with more light for deceleration may permit an injection speed of 14.9%c.) If the graphene had holes, as suggested for other materials (Atwater *et al.*, 2018), then the mass of the sail might be twofold less, and the velocity higher than by a factor of 2^0.5^.

Although it is not yet certain how to deploy 10^5^ m^2^ atomically thin sails, 3000-fold smaller sail (0.25 mg double-layer graphene-on-copper) scales have been tested in vacuum and in microgravity using 450 and 655 nm lasers at 0.1–1 W (Gaudenzia *et al.*, 2020). Indeed, these experiments observed an 8–37 nN thrust for a light power of 0.1 W, while a maximum of ∼0.4 nN comes from theoretical calculations for radiation pressure alone. Depending on the explanation of this disparity, the additional factor of at least 20-fold might further encourage us to consider speeds more than 20^0.5^-fold higher.

An alternative to launching picogram probes from the Earth and decelerating based on starlight would be decelerating from a subgram-scale flyby craft. If 2 × 10^11^ W is recommended for a 3.6-g (4 m sail) probe (Parkin, 2018), then 0.5 W might suffice for an 8 × 10^−12^ g probe. Parkin noted that (hypothetically) the craft “transmits 100 Watts, deriving its power from a 700-Watt hydrogen beam that is normally incident on the sailcraft … simply a manifestation of the interstellar medium” (Parkin, 2020). The redirection of many of the functions of the larger probe to the smaller probes might permit a 100-fold smaller, faster, and/or less costly mothership (40 mg, 0.4-m-diameter sail) and 2 GW Earth launcher.

It has been noted (Heller and Hippke, 2017) that a sail with 99.99% reflectivity absorbs 242 W/m^2^ corresponding to an effective temperature of 256K. T = (p/e*σ*)^0.25^, where the power per unit area absorbed p = P(1 − R), e = emissivity (*e.g.,* 0.8), and *σ* = the Stefan–Boltzmann constant (5.67 × 10^−8^ W/m^−2^ T^4^) (Kim *et al.*, 2013). Although the context above is graphene, which is stable to 4000K, a microbial payload temperature maximum is 300K to 693K, and so, if a lower reflectivity should be used, the payload should be protected and distanced from the sail.

Although a gram-scale probe is only intended as a flyby, one can consider the consequence of landing from 0.2c with kinetic energy, mc^2^((1 − (v/c)^2^) ^−0.5^ − 1) = 1.9 × 10^12^ J ( = 1/8th the 1945 Hiroshima 235U bomb). In contrast, a 0.2c nanogram landing would be 2 kJ ( = 0.5 food calorie). *En route* from our solar system to another, a probe may encounter dust grains (typically <500 nm) at a rate of 10^−8^ (100 nm) to 10^−6^ m^−3^ (10 nm) and hydrogen atoms at 2 × 10^5^ m^−3^ (Ferriere, 2001; Lubin, 2016; Siegel, 2022). Traveling the full 4 × 10^16^ m with a cross-sectional area of 0.5 × 10^−10^ m^2^ (8-μm diameter) would mean collisions with 10^−12^ g of atoms and two dust particles on average.

Collisions with dust are likely fatal to the small probes, with a Poisson-distributed survival rate of 13% (however, the mean = 2 is a soft estimate, and the hit rate is an exponential of it). The momentum changes due to collision with hydrogen, integrated on the entire multiyear journey, would be small (0.1%), so the bulk of deceleration would rely on photons from stars. Yet collision with a single proton could release 3.2 × 10^−15^ J (6000-fold more than a covalent bond) and the ensuing erosion could affect the sail reflectance and/or performance. The scenario above with a 10^−8^ m^2^ graphene sail could mean 200-fold more hydrogen atom collisions, hence encouraging a design in which the larger (solar) sail is deployed only at the end of journey, possibly released by erosion of the original small spherical (laser) sail.

## 5. Building Communication Devices

To “phone home,” we need some form of electromagnetic radiation, which is brighter than the resolution-limited surroundings for at least some wavelength and for a short time frame.

The message contents would include parameters to optimize subsequent probes from the Earth.

Some data pre-encoded at launch: angle (right ascension and declination), materials, velocity, and other parameters describing the destination planet: temperature, pressure, pH, environmental chemistry, and images. Synthetic biology is exponentially improving in ability to programming photon outputs coupled to a variety of biosensor inputs (Zhou *et al.*, 2020). The communications “device” could be constructed and aimed using engineered organisms through two main strategies: (1) planetary-scale bioluminescence or (2) conversion of starlight through a laser to a narrow and/or shifted wavelength using frequency doubling or Stokes shift. Method (1) is conceptually far simpler and much closer to a demonstration project, but more invasive. Each method is considered below.

### 5.1. Bioluminescence

A recent study (Tabor and Loeb, 2021) estimates that the James Webb Space Telescope is able to detect current Earth levels of artificial illumination on Proxima b, if the spectral band were 10^3^ times narrower. Whether or not this is considered plausible for natural lighting, this might be engineered through bioluminescence. Luciferase enzymes have already been engineered for enhanced stability, smaller size, more than 150-fold increase in luminescence over natural luciferase enzymes (England *et al.*, 2016), and wavelengths from blue to near-IR (449–662 nm) (Tamaki *et al.*, 2021). Similar improvements have been made for fluorescent proteins originally derived from jellyfish and corals. For example, dinoflagellate marine photoautotrophs produce 475 nm light at night (Valiadi and Debora Iglesias-Rodriguez, 2013), with flashes ranging from 80 ms in *Noctiluca scintillans* to 150 ms in *Lingulodinium polyedrum* and 500 ms in *Pyrocystis fusiformis* (at 10^7^ photons per cell in *Gonyaulax excavata* to 10^9^ photons per cell in *Pyrocystis noctiluca*).

The flashes can be synchronized over any volume, for example, by shared pressure pulses (*e.g.,* from delighted human swimmers). *Photinus carolinus* (US Smoky Mountain fireflies) synchronize light pulses in waves, while *Pteroptyx* (SE Asia) are more completely simultaneous. These can be coordinated among many separate organisms throughout entire trees but doing so over a planetary hemisphere requires additional engineering. Signal coordination within a single organism can be faster and more faithful. Delays and feedback can be built in (as in neural circuits) to achieve higher levels of synchrony (rather than waves). *Omphalotus*, *Armillaria*, and *Mycena* genera include dozens of luminescent species. The largest known organism on the Earth is said to be *Armillaria ostoyae*, a 10 km^2^ fungus.

The fastest doubling time for an autotroph (*e.g.,* algae) is 1.4 h, which (given no predators, ideal conditions, and mixing) could monolayer coat a 5 × 10^8^ km^2^ planetary surface in 1.4 × log_2_(5 × 10^26^) = 124 h. Many biomolecules (*e.g.,* lipids at 40 MJ/kg) have 50-fold higher energy density than common electric storage devices (0.8 MJ/kg for Li-ion batteries). Short pulses of a particular bioluminescent wavelength could be brighter than the sum of human night lights since only 1% of Earth (3% of land) is urban and much of that light from cities is absorbed indoors or by vegetation.

Synthetic biologists have designed and optimized “ring oscillators” composed of three repressor proteins to enable biocircuits, which “kept phase for hundreds of generations in single cells, cells in flasks and colonies oscillate synchronously without any coupling” (Potvin-Trotter *et al.*, 2016). Each cycle can take about 8 h. This could be used to coordinate organisms on the scale of a pond, sea, or atmosphere in which a single spontaneous flash from one cell could trigger the rest to initiate. A monotonous series of nucleotide pulses can be converted to an information-rich series of bioluminescent pulses programmed by a DNA sequence in a process called pyrosequencing (Ronaghi, 2001).

A polymerase enzyme elongates a primer bound to a long DNA template only if the correct nucleotide monomer is present (dGTP if the next base in the template is “C”; dATP for “T,” dCTP for “G,” and dTTP for “A”). Only if the elongation reaction occurs, an inorganic pyrophosphate (PPi) molecule is liberated and the following two reactions occur (over and over, until reset):

$$PPi+APS\to ATP+Sulfate\left(catalyzedbyATP-sulfurylase\right);$$

$$\begin{array}{cc} & ATP+luciferin+O_{2}\to AMP+PPi+oxyluciferin+CO_{2}\\ & +h\nu \left(catalyzedbyluciferase\right); \end{array}$$

The PPi produced start the next step of the cycle, producing more photons (hν) and more PPi. If the monomer is incorrect, then no light is produced. Another enzyme called apyrase resets the reaction for a another try in order A,C,G,T, A,C,G,T … These reactions can stay synchronous (among billions of molecules) for about 600 cycles of 1 min per cycle, at which point, the whole process can be reset by the next step in the aforementioned ring oscillator. The enzymes and nucleotide substrates would likely be altered in shape so as to not interfere with the rest of the cell metabolism. The production of the monotonous series of nucleotide monomers (dGTP, etc.) would require a cycle of its own.

The DNA sequence used for pyrosequencing above could be selected from various prerecorded DNAs (*e.g.,* metabolic or launch parameters) or environmental nucleic acids (if any) encountered, or molecular recordings can be made in response to environmental factors by harnessing a template-independent DNA polymerase enzyme (typically the TdT; Lee *et al.*, 2019, enzyme found in nature generating antibody gene diversity in our B cells). This has been shown for recording concentrations of a hypoxia mimic chemical in living cells (Loveless *et al.*, 2021). Hopefully there will emerge many ways to accomplish the programming of these light pulses, brighter, higher bandwidth, and so on. This is just intended as a proof of concept that at least one such system might be feasible.

### 5.2. Biolaser

To permit a smaller footprint than the above scenario for a communication device, a laser can create distinctive light, for example, through a narrow linewidth (1 kHz = 0.1 nm), yielding perhaps a 10,000-fold enhancement. Potassium dihydrogen phosphate is used for frequency doubling from 1064 to 532 nm or from 795 to 397 nm, which could be another factor of 10 to 100 improvement in background light. Solar “compound parabolic concentrator” designs have large acceptance angles and elimination of a tracking requirement. They are named after their seashell shape (Tian *et al.*, 2018), which could be combined with highly reflective biomaterials (see table below). Pearl nacre reflectance ranges from 80% to 90% in a broad range of wavelengths (at least 300–800 nm) (Mamangkey *et al.*, 2010).

The gold beetle *Aspidomorpha tecta* grows its own “chirped” stack reflector, refractive indices alternating between 1.73 and 1.40 yielding 91% reflectance (Parker *et al.*, 1998; Wang *et al.*, 2021). As an example (Kong *et al.*, 2005), an 8% Yb:Y_2_O_3_ ceramic laser converts a 27-W 937 nm pump light to an output of 1078 nm at 9.22 W, the pump light focused to a 0.45 mm spot on the 3 mm ceramic. The laser cavity is bounded by an input mirror with a highly reflective (1030–1100 nm) coating and antireflective (937 nm) coating and a 1000-mm radius of curvature (ROC). The output mirror has 96% reflectivity at 1064 nm and a 50 mm ROC. Making a biofabricated version of this would be an interesting laboratory challenge.

## 6. Environments for Replication with Suitable Temperature and Chemicals

Minimal elements for replication of current microbes include C, H, O, N, P, S, Mg, K, Na, Ca, and possibly Mn, Fe, Co, Cu, Zn, Mo. Eliminating a few of those would be a challenging synthetic biology project using accelerated laboratory evolution. Replicating buoyant structures in planetary atmospheres could be an option if ground temperatures or other parameters are extreme (see discussion of Uranus and Neptune below). Living systems often transport molecules against steep concentration gradients. For example, a cyanobacterium isolate from the hyperarid Chilean Atacama Desert generates biofilms that induce water extraction from gypsum rock (CaSO_4_·2H_2_O) with phase transformation to anhydrous calcium sulfate (Huang *et al.*, 2020). *Halobium salinarum* grows optimally in nearly saturated NaCl solutions and maintains an intracellular K+ concentration 110 times that of the medium.

Often cells concentrate molecules past the point of solubility, thus forming crystals (see table below)—generally stabilized mechanically by sparse, precise biopolymer strands. Engineering such extreme concentration gradients is a common task in metabolic engineering.

## 7. Biomaterials with High Reflectance or Other Utility

Crystal morph variants in parenthesis.

**Table:** 

Aragonite (orth), calcite (trig), vaterite (hex) CaCO_3_	Mollusc shells, *Bacillus cohnii*
(Hydroxy)Apatite Ca_5_(PO_4_)_3_OH	Bones, teeth, *Providencia rettgeri*
Fluorapatite Ca_5_(PO_4_)_3_F	Teeth
Celestine SrSO_4_	*Acantharia*
Fluorite CaF_2_	Inner ear
Lepidocrocite, goethite γ,αFeO(OH)	Limpet teeth*, Acidovorax*
Magnetite, greigite Fe_3_O_4_, Fe^2+^Fe^3+^_2_S_4_	Magnetotactic bacteria, vent snail
Perovskite BaTiOF_4_	Sponge *Tethya aurantia*
Pyrite (cubic), marcasite (orth) FeS_2_	Sulfate-reducing bacteria, vent snail
Quartz SiO_2_	Sponge spicules, diatoms
Whewellite Ca C_2_O_4_·H_2_O	Plants and fungi
Reduced metals Ag, Au, Pd, Pt	Microbes and plants (Shah *et al.*, 2015)
Metal sulfides/oxides CdS, ZnS, In_2_O_3_, Sb_2_O_3_	Microbes and plants (Shah *et al.*, 2015)

Often materials that humans have only made with high temperatures and/or pressures are made biologically at 4° and Earth atmosphere (Müller *et al.*, 2008). High concentrations can be achieved through protein binding and transmembrane pumps with high molecular specificity and avidity. Even isotope pairs such as ^2^H/^1^H and ^12^C/^13^C are discriminated by enzymes. Recently, engineering protein specificities advanced significantly with the use of machine learning plus mega-libraries (Bryant *et al.*, 2020). Relevant to the laser mentioned above, *Daphnia magna* sequesters up to 75% of yttrium (Cardon *et al.*, 2019), and the *Escherichia coli* Dps protein sequesters ytterbium (Zeth *et al.*, 2021).

Examples of organisms adapted to desiccation and, as a by-product of this, to radiation include *Polypedilum vanderplanki*, an arid midge surviving 3K to 100°C, 7 kGy γ, and the Tardigrada, broadly distributed, 1K to 147°C, median lethal dose of 5 kGy hydrated. It has been shown that as few as 4 genes can convert a radiation-sensitive species into a highly resistant one (100,000-fold higher) (Bruckbauer, 2020). It should be noted that, although DNA damage due to radioactive decay (*e.g.*, ^14^C or ^40^K) will be less due to relativistic time dilation, damage from external sources (galactic γ photons) could be a bit worse due to blue shifting—and in any case, worse than inside a warm spaceship, because normal biochemical repair processes are prevented by low temperatures. *Deinococcus* bacteria withstand 15 kGy, *Picrophilus* pH 0, and *Bacillus* PB12 pH 12.

## 8. Temperature and Energy Sources

Temperatures enabling microbial replication found so far range from −20°C to +122°C (a subset of liquid water −42°C to 374°C). Up to 420°C is survivable by dry spores of *Bacillus amyloliquefaciens* (Beladjal *et al.*, 2018). The temperature range for extreme electronics and electromechanical systems is 3K to 700°C, but devices using such components are not nanogram scale nor capable of replication from only simple chemicals. Some planets tidally locked to their star may only have liquid water at a narrow, fixed terminus line between light and dark making for a hard target to hit. Some planets and moons may have considerable liquid water but are locked under a surface layer of ice recalcitrant to penetration by nanogram-scale objects, for example, 20 to 800 km thick on Europa and Ganymede in our solar system.

Proxima b lies in a “habitable temperate zone” but receives only 3% of the photosynthetically active wavelengths of Earth and potentially 10,000-fold more atmosphere-stripping flares (and if tidally locked then a very thin strip of liquid water). The light spectrum from Proxima b star is at the red edge of common terrestrial plant (BChl)-based anoxygenic photosynthesis. Proxima Centauri b has low irradiance in the oxygenic photosynthesis range (400–749 nm), while the anoxygenic bacteriochlorophyll extends to 1100 nm. Ritchie *et al.* (2018) found “photosynthesis on Proxima Centauri b could be surprisingly high (oxygenic photosynthesis: earth ≈0.8 gC/(m^2^·h); Proxima Centauri b ≈ 0.14).”

Photoautotrophy is not the only way for converting energy to build large organic molecules from carbon dioxide and other inorganic components. Chemolithoautotrophic archaea and bacteria are found in the deep ocean (up to 1 kbar = 100 MPa pressure) with some metabolism at 0°C and some thriving at 122°C near hydrothermal vents. Alpha Centauri A and B are Sun-like stars (Class G and K), and so, if a planet such as Candidate1 (aka C1 or Alpha Centauri Ab) is confirmed (Wagner *et al.*, 2021), it might be more suitable in some ways (above) plus potential for a planet-wide layer of “water–ammonia ocean” as may occur on Uranus and Neptune—but less suitable if no combination of temperature, pressure, and so on is in the range for life (despite, *e.g.,* 55K to 5400K gradient), or if outgoing light is inadequately transmitted to space.

## 9. Orientation Precision for Launch and Reply

As mentioned above, we might be able to afford trillions to quadrillions of nanogram or picogram probes.

A few groups have explored the use of spherical shell design (Manchester and Loeb, 2017; Atwater *et al.*, 2018) for a light sail capable of stable beam riding without the need for active feedback control. “A sum of four Gaussians … appropriately sized spherical sail perturbed from the center of such a composite beam will experience a restoring force pushing it back toward the center.” Diffraction-limited divergence angle is given by θ = 2.44λ/D = 7 × 10^−7^ radian (for λ = 1 μm, D = 4 m). Since Alpha Centauri A is 4.1 × 10^16^ m from the Earth, the optical error will be 3 × 10^9^ m, similar to the diameter of our Sun (1.4 × 10^9^ m). This applies in both directions.

In addition, on the way out, roughly 10^12^ proton collisions, random walk distributed directional offsets, each having a 10^−15^-fold effect. In the return message to Earth, additional issues arise due to the (nonhuman) orientation of the laser.

How to provide detailed, genetically encoded instructions of absolute shape and orientation is a rapidly progressing subfield of synthetic biology. Although mushrooms (and other fungi) have intriguing potential for precise shape and orientation, few or no engineering studies have optimized this. Some fungi form upward-facing flat or parabolic cups, for example, Elfcup (*Sarcoscypha austriaca*) and Funnel cap (*Clitocybe gibba*). Bioengineering is in the midst of a revolution in harnessing morphological specifications from genomes, including cellular and molecular feedback on the shape and function (Gramelsberger, 2020; Ebrahimkhani and Levin, 2021). The fungus *Dictyophora indusiata*, one of the world's fastest-growing organisms, extends upward at a rate of 0.5 cm/min. *Ascobolus immersus* catapults spores with an acceleration of 180,000 × Earth gravity. The smallest basidiospores are produced by *Hyphodontia latitans* with a mass of 0.6 pg. Dispersal of spores while landing might increase the odds of finding adequate growth conditions.

## 10. Biocontainment and Biocontamination

A 2018 NASEM report on the Planetary Protection Policy (NASEM, 2018) said that “DNA sequencing techniques may be able to identify organisms that come from Earth-bound spacecraft assembly cleanrooms without the need to treat every microbe on a spacecraft as a potential compromising agent … NASA should engage the full range of relevant scientific disciplines in the formulation of its planetary protection policies. This requires that scientific leaders outside of the standard planetary protection community in NASA participate.” The utility of probes having biological components (sensitive to sterilization protocols), such as nanopore sequencing proteins, has been discussed, as has the potential for intentional inclusion of replicating components (as in von Neumann probes).

The issue of biocontainment merits additional study. As one example, microbes have been made completely dependent on a nonstandard amino acid (NSAA), present in a tiny, but essential, fraction of cell's biopolymer molecules (Mandell *et al.*, 2015). The key NSAA is typically 1/200th of the protein mass, and that protein can constitute <0.01% of the mass of a cell (Ishihama *et al.*, 2008), and a cell can assimilate and secrete extracellular biofilms much larger than the cell mass. Essential NSAA molecules are engineerable to any amount less than that if that cell is required for the replication of nearby cells. One can also engineer cell circuits to count the number of cell divisions or the quorum density and then stop (permanently).

## 11. Summary

**Table:** 

Proposed feature	Potential advantages	Major challenges
Gram to pg	Capex: × 10^−12^, Landing	Damage *en route*, water
Gram+pg	Landing	Small impact on min capex
Graphene sail	Solar deceleration	Exceeding 4.6%c, damage
Luminescence	Distinct λ, size: × 10^14^	Synchrony, bandwidth, km^2^ extent
Biolaser	Small footprint, focus: × 10^7^	Programming shapes and orientation

Clearly, a considerable amount of work remains for improving the theory, design, and testing aspects of this proposal, some of which can be done on the Earth (Gaudenzia *et al.*, 2020) or within our home solar system (*e.g.,* Uranus or Neptune)—including deceleration, landing, replication, and bioconstructed communicator. Obviously, this is a speculative, multidisciplinary proposal limited by a single author (although gratefully leveraging recent Starshot discussions). Suggested improvements or exposures of fatal flaws are welcome.

## Notes

Associate Editor: Christopher McKay

## Abbreviations Used

IR: infrared
NSAA: nonstandard amino acid
PPi: inorganic pyrophosphate
ROC: radius of curvature
